# Mechanochemical Transformation From Zigzag‐Type Layered/Phenakite to Disordered Rocksalt in Mn‐Rich Cathodes for Li‐Ion Batteries

**DOI:** 10.1002/smll.73666

**Published:** 2026-05-07

**Authors:** Yi‐Chen Wu, Yoojin Ahn, Tsung‐Yi Chen, Xueyu Hu, Yong Ding, Yewon Oh, Weining Wang, Meilin Liu

**Affiliations:** ^1^ School of Materials Science and Engineering Georgia Institute of Technology Atlanta Georgia USA; ^2^ National Synchrotron Radiation Research Center Hsinchu Taiwan

**Keywords:** cathode, disordered rocksalt, lithium‐ion battery, mechanochemical synthesis, phase transformation

## Abstract

Understanding how cation disorder develops in Mn‐rich disordered rocksalt (DRX) systems—particularly under mechanochemical activation—has been hindered by limited insight into the underlying transformation pathways. Here, we uncover a distinct transformation route in Li_1.2_Mn_(2+x)/3_Mo_(0.4‐x)/3_O_2‐x_F_x_ (LMMOFx), in which Mo and F co‐doping drives the material toward a zigzag‐type layered/phenakite‐mixed structure. This layered/phenakite‐mixed phase undergoes pronounced local structural reconstruction, which promotes DRX formation and induces a transition from a two‐phase Mn and O redox mechanism to a stable single‐phase process, delivering a high reversible capacity of 297.9 mAh g^−1^. Systematic structural and electrochemical comparisons across the LMMOF series further reveal how progressive disorder and phase mixing reshape Li‐ion transport pathways. Together, these results establish a mechanistic framework for leveraging dopant‐ and structure‐controlled precursor chemistry to direct mechanochemical DRX formation, and they offer a design principle for earth‐abundant, Mn‐rich DRX cathodes with tunable disorder.

## Graphical abstract

Mechanochemical activation drives the transformation of a Mo/F‐modified layered/phenakite precursor into a Mn‐rich disordered rocksalt phase. The resulting disordered rocksalt structure improves cation mobility and structural reversibility during cycling, enabling higher reversible capacity and enhanced electrochemical performance.

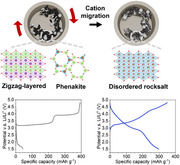

## 1 Introduction

Among various cathode materials for lithium‐ion batteries (LIBs), olivine, spinel, and layered oxide represent the three predominant structural families [1, 2, 3]. However, the most widely adopted commercial O3‐type layered (*R‐3m*) cathode materials, such as LiCoO_2_ and LiNi_0.8_Mn_0.1_Co_0.1_O_2,_ depend heavily on costly and scarce transition metals (TMs) like Co and Ni [4, 5, 6, 7, 8]. This reliance not only increases production costs but also limits the scalability of large‐scale deployment [9, 10]. As global demand for LIBs continues to grow, the development of energy storage materials based on earth‐abundant TMs is becoming increasingly important to enable sustainable, next‐generation technologies [11, 12].

Cation‐disordered rocksalt (DRX) (*Fm‐3m*) structures have been studied as a promising class of cathode materials, offering high capacity and the use of earth‐abundant TMs, thereby meeting the requirement of low‐cost and sustainable next‐generation LIBs [13, 14]. Unlike traditional ordered structures, where cations occupy well‐defined lattice positions, DRXs feature randomly distributed cations, reducing the necessity of specific TMs that promote cation ordering [15, 16]. However, the formation of DRX structures generally requires high local temperature and pressure to enable structural rearrangement from ordered rock salt structures, which can increase production costs [17, 18, 19].

In addition to cation redox, DRX cathodes can deliver higher capacity than conventional cathodes by utilizing anion redox [20, 21]. However, despite this advantage, DRX materials suffer severe cycling degradation caused by anion loss during repeated anion redox processes at high voltages [22, 23]. To mitigate this issue, researchers have investigated strategies such as tuning redox‐inactive TMs and incorporating F doping to suppress irreversible anion redox and enhance structural stability [24, 25, 26, 27, 28]. Nevertheless, the impact of these approaches on the structural evolution of DRX during mechanochemical synthesis remains unclear. Furthermore, the electrochemical behavior, capacity decay, and structure change mechanism of DRX are still not fully understood, highlighting the need for deeper insights and continued research to unlock the full potential of DRX materials.

In this research, we designed Li_1.2_Mn_(2+x)/3_Mo_(0.4‐x)/3_O_2‐x_F_x_ (LMMOFx) to explore the influence of Mo and F on electrochemical performance and structural formation of Mn‐rich DRX cathode materials. We introduce Mo and F as essential dopants on Mn‐rich compositions to induce a mixed‐phase of zigzag‐layered (*Pmmn*) and phenakite (*R‐3*) (layered/phenakite) structures, facilitating DRX mechanochemical formation during ball‐milling. This finding provides new mechanistic insights into how mechanical milling influences cation disorder, phase transformation, and lithium‐ion transport in DRX lattices. Unlike conventional DRX synthesis routes that rely on direct mechanochemical disordering or post‐synthesis milling, this work employs a deliberately designed layered/phenakite‐mixed precursor to facilitate the subsequent transformation. This strategy enables more efficient DRX formation by lowering the cation disordering barrier through enhanced intrinsic cation mobility. Our research reveals a novel transformation pathway from a layered/phenakite‐mixed phase to a DRX structure, driven by local heat and pressure and validated through X‐ray characterizations, Raman spectroscopy, and electron microscopy. As a result, the high electrochemical performance (297.9 mAh g^−1^) can be achieved through this transformation route, mitigating the reliance on the use of rare transition metal dopants. Furthermore, this work advances fundamental understanding of phase evolution and structure‐electrochemistry relationships in Mn‐rich DRX systems, establishing a new design strategy for Co/Ni‐free and earth‐abundant cathode material development with tunable disorder and enhanced electrochemical performance.

## 2 Results and Discussion

### 2.1 Effect of Mo and F Co‐Doping on Mechanochemical Transformation

**FIGURE 1:**
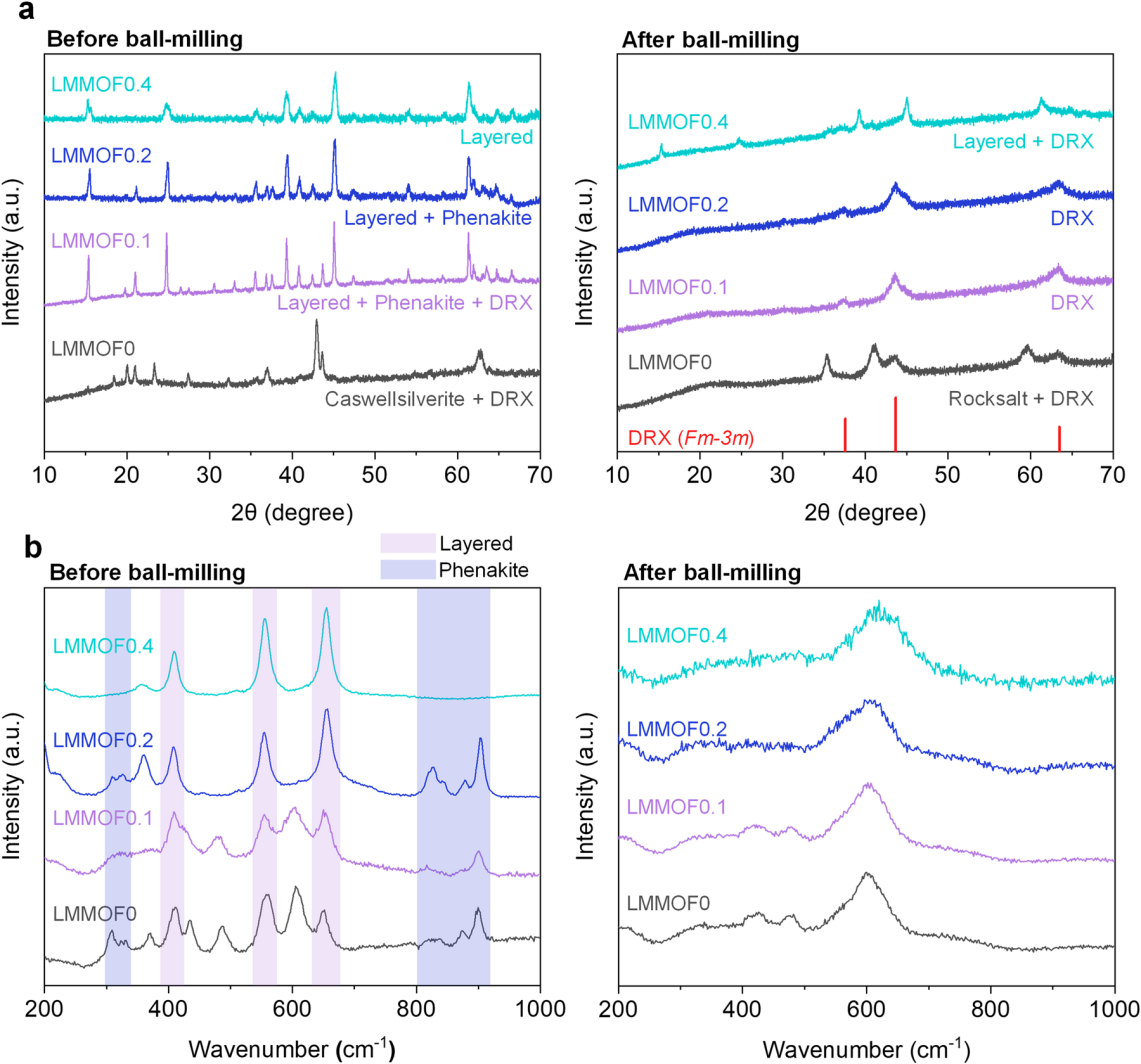
Structural characterization of LMMOF samples. (a) XRD patterns and (b) Raman spectra before and after ball‐milling. All samples were ball‐milled for 4 h at 450 rpm.

To elucidate the role of Mo and F co‐doping in the formation and stabilization of the DRX structure, X‐ray diffraction (XRD), Raman spectroscopy, X‐ray photoelectron spectroscopy (XPS), and X‐ray absorption spectroscopy (XAS) were employed to characterize the materials before and after the ball‐milling process. XRD analysis reveals the structural evolution of the four DRX materials with varying Mo and F contents (LMMOFx, *x* = 0, 0.1, 0.2, 0.4), as shown in Figure 1a, Figure S1, and Note S1. Before mechanical treatment, the Mo and F co‐doped materials—LMMOF0.1 and LMMOF0.2—mainly exhibit a layered/phenakite‐mixed phase, with LMMOF0.1 also containing a minor DRX component. Supporting thermogravimetric analysis (TGA), differential scanning calorimetry (DSC), and XRD data for LMMOF0.2 indicate that the phenakite phase forms at a higher temperature than the layered phase, emerging above ∼700°C (Figure S2). This suggests that the layered and phenakite phases are thermodynamically competitive under the synthesis conditions, while the persistence of the layered/phenakite‐mixed phase indicates that complete phase equilibration is kinetically limited, likely due to restricted cation diffusion during calcination. Following ball‐milling, both the layered and phenakite phases disappear, accompanied by the emergence of broad diffraction peaks characteristic of the DRX phase. This demonstrates the complete transformation of the layered/phenakite‐mixed phase structure into a DRX phase. In LMMOF0, which does not contain F, the material evolves from a Caswellsilverite‐type structure into two coexisting rocksalt phases: a Li‐containing DRX phase and a MnO‐based rocksalt phase [29]. Because these phases have different Li contents, their lattice parameters differ, leading to XRD peaks at distinct angles. LMMOF0.4, which does not contain Mo, initially exhibits a layered structure, and after ball‐milling, it contains both the layered and DRX phases. These observations suggest that Mo and F dopants act synergistically to lower the energy barrier for DRX formation, facilitating the mechanochemical transformation from the layered/phenakite‐mixed phase to the disordered phase under ball‐milling conditions with identical local pressure and heat.

Furthermore, bonding structures were characterized by Raman spectroscopy (Figure 1b; Figure S3). Before ball‐milling, LMMOF0.2 exhibits prominent Mo‐O Raman bands at ∼320, ∼850, and ∼900 cm^−1^, corresponding to the bending, asymmetric stretching, and symmetric stretching modes of Mo‐centered tetrahedra in the phenakite‐type Li_2_MoO_4_ structure, respectively [30]. LMMOF0.2 also exhibits Raman bands at ∼440, ∼550, and ∼650 cm^−1^, which are generally assigned to the Mn‐O bending, asymmetric stretching, and symmetric stretching modes of Mn‐centered octahedra in the layered‐type LiMnO_2_ structure, respectively [31]. LMMOF0.4 primarily shows Raman band characteristics of the layered structure. In contrast, LMMOF0.1 and LMMOF0 show TM‐O bending and stretching modes at ∼490 and ∼600 cm^−1^, respectively, rising from the octahedra in the DRX structure [32]. After ball‐milling, LMMOF0.2 shows a dominant TM‐O stretching mode around 600 cm^−1^, while the TM‐O bending modes become significantly weakened due to the combined effects of cation disorder and local symmetry relaxation in the DRX structure, which reduces the Raman activity of bending vibrations [33, 34]. Together, XRD and Raman analyses confirm that Mo and F co‐doped LMMOF0.2 predominantly transformed from a layered/phenakite‐mixed phase to a DRX phase upon ball‐milling.

**FIGURE 2:**
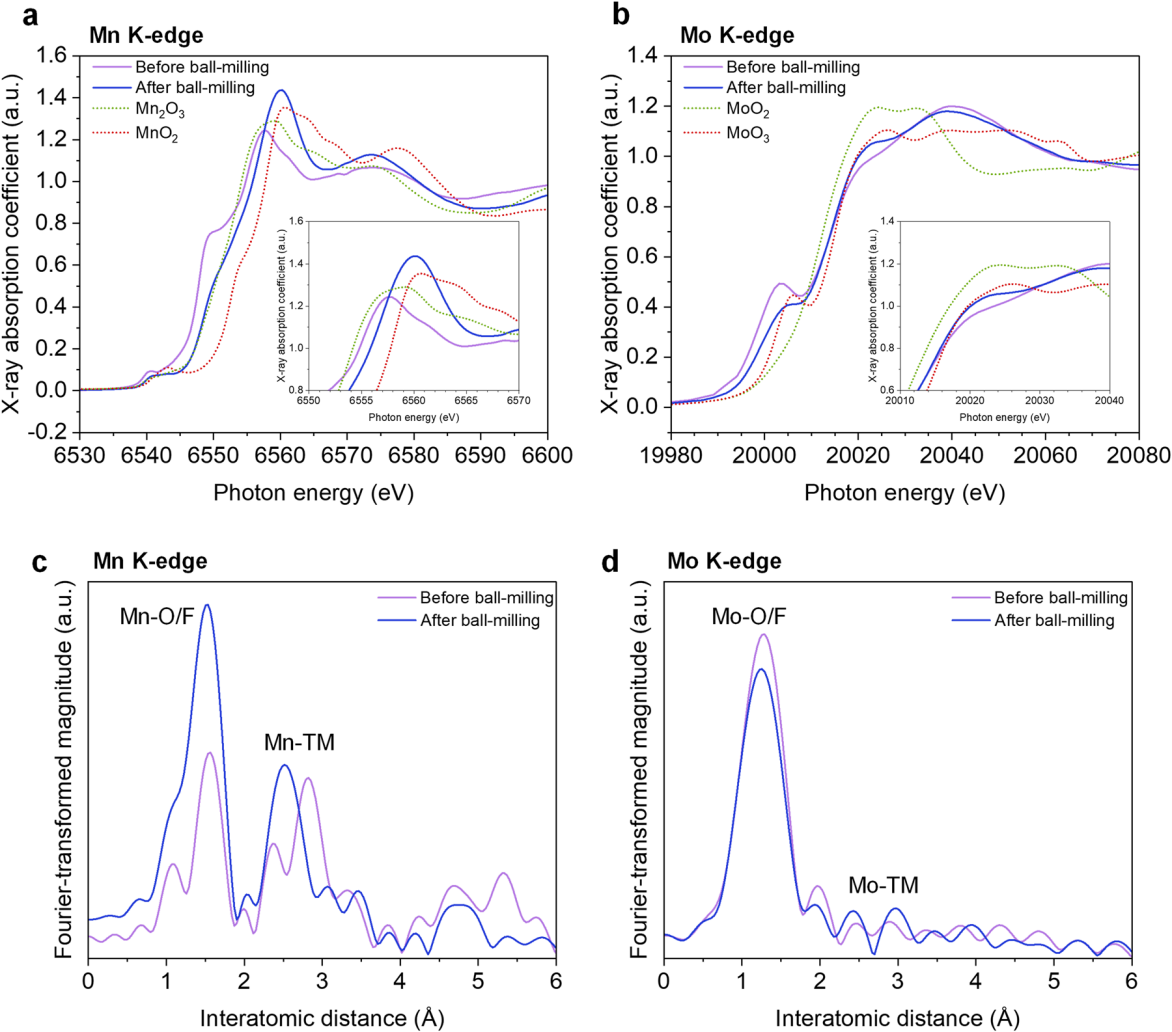
X‐ray absorption spectroscopy of LMMOF0.2. XANES of (a) the Mn K‐edge and (b) the Mo K‐edge before and after ball‐milling. EXAFS of (c) the Mn K‐edge and (d) the Mo K‐edge before and after ball‐milling.

The mechanochemical phase transition from the layered/phenakite‐mixed phase to the DRX phase also affects the oxidation states of the transition metals. X‐ray absorption near‐edge spectroscopy (XANES) and XPS analyses show that LMMOF0.2 mainly contains Mn^3+^ with a minor fraction of Mn^4+^, and the average Mn oxidation state slightly increases after ball‐milling (Figure 2a; Figures S4, and S5). Similarly, LMMOF0.2 initially contains Mo^6+^ with a minor amount of Mo^5+^, which primarily converts to Mo^6+^ after ball‐milling (Figure 2b; Figures S6, and S7). It should be noted that XANES primarily reflects the bulk electronic structure, whereas XPS is more surface‐sensitive. Nevertheless, both techniques consistently indicate an increase in oxidation state after ball milling, suggesting that the oxidation is not confined to the surface but extends throughout the material. XPS measurements also reveal both TM─F and TM─O─F bonds before ball‐milling, whereas the TM─O─F component is significantly diminished afterward (Figure S8a). This suggests that the mechanochemical process promotes a shift from TM─O─F environments to more direct TM‐F coordination, enabling fluorine to more effectively withdraw electron density from the transition metals [35]. Such electron redistribution may contribute to the observed increase in oxidation state and is consistent with the reduction of non‐lattice oxygen (Figure S8b).

The extended X‐ray absorption fine structure (EXAFS) analysis further confirms the lattice and bonding rearrangement induced by the mechanochemical phase transition (Figure 2c,d; Figure S9). In the Mn and Mo K‐edge spectra, the Mn‐O/F, Mn‐TM, and Mo‐O/F interatomic distances decrease by 2.0%, 10.9%, and 4.8%, respectively. The Mo‐TM signal is not clearly resolved due to the relatively higher atomic number of Mo and overlapping scattering contributions. The formation of shorter TM─F bonds relative to TM‐O partially explains the reduction in average TM‐O/F distances. Notably, the 10.9% decrease in Mn‐TM distance is well supported by the EXAFS fitting (11.0% decrease in Mn‐TM distance with R‐factor < 0.02), indicating that the observed change is not an artifact of model selection but reflects a genuine structural evolution (Figure S10). This reduction is attributed to lattice rearrangement driven by cation disorder and local structural distortions in the DRX structure, rather than a simple geometric contraction [36]. Additionally, the replacement of TM─O─TM linkages with TM─F─TM linkages may contribute to the observed reduction in Mn‐TM distances in the EXAFS results.

**FIGURE 3:**
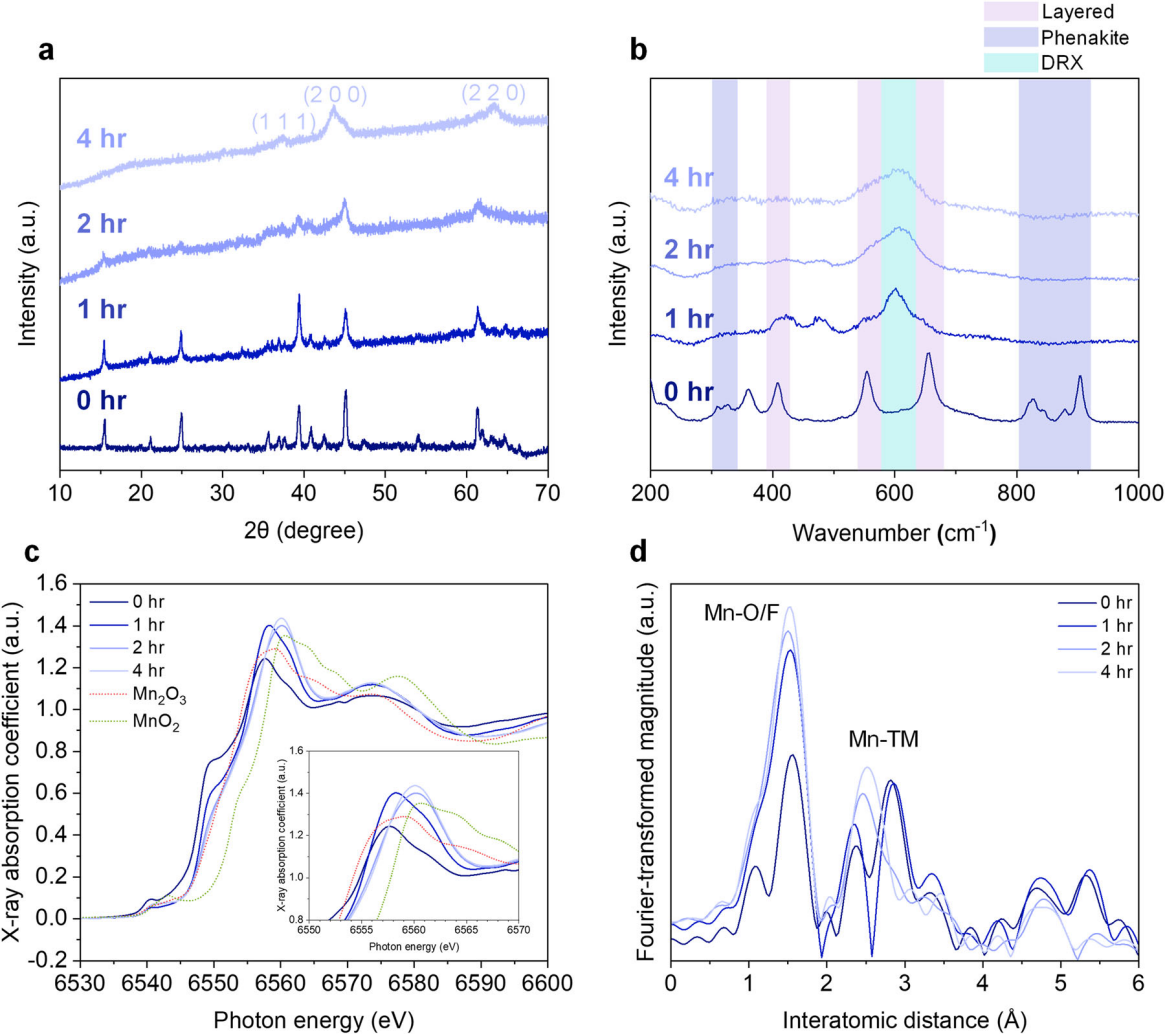
Structural and electronic characterization of LMMOF0.2 at different ball‐milling durations. (a) XRD, (b) Raman spectra, (c) XANES, and (d) EXAFS after 0, 1, 2, and 4 h of ball‐milling.

To elucidate the mechanochemical transformation of LMMOF0.2 from a layered/phenakite‐mixed phase to a DRX structure, we performed a series of characterizations during ball‐milling. XRD patterns collected at different milling durations show a gradual structural transition from the layered/phenakite‐mixed phase to the DRX phase (Figure 3a). As milling progresses, the peaks associated with the Mn‐based layered and Mo‐based phenakite gradually weaken and broaden, indicating increasing lattice strain and particle size reduction. The layered structure remains discernible after 2 h of milling. After 4 h, only three peaks at 37.4°, 43.7°, and 63.5°, corresponding to the (111), (200), and (220) reflections of the DRX structure, remain [37, 38]. Further milling up to 8 h produces no additional changes, confirming that 4 h of milling are sufficient to achieve complete DRX formation (Figure S11). Raman spectroscopy also tracks this transformation. The Raman bands associated with the layered and phenakite phases diminish and disappear within 1 h of milling, while a new band near 600 cm^−1^ emerges, corresponding to TM‐O stretching in the DRX phase (Figure 3b). This rapid change in Raman signals, relative to the slower disappearance of layered and phenakite reflections in XRD, suggests that DRX formation initiates at the particle surface during ball‐milling. The local bonding environment and particle size reduction induced by ball milling may also influence the Raman scattering behavior. As the particle size decreases to the nanoscale, nanoparticles with a distribution of sizes may exhibit slightly different vibrational frequencies, and the superposition of these contributions can lead to Raman peak broadening and reduced intensities. The Mn and Mo oxidation states also increase slightly with milling (Figure 3c; Figure S12). EXAFS analysis shows interatomic distance changes consistent with the XRD measurements; notably, the Mn‐TM distance shifts significantly between 1 and 2 h, coinciding with the rapid decrease of the layered structure (Figure 3d). The mechanochemical formation is also influenced by rotational energy during ball‐milling, where higher milling energy at increased rotation speed promotes more efficient DRX formation (Figure S13).

### 2.2 Microscopic Observation of Mechanochemical Transformation

**FIGURE 4:**
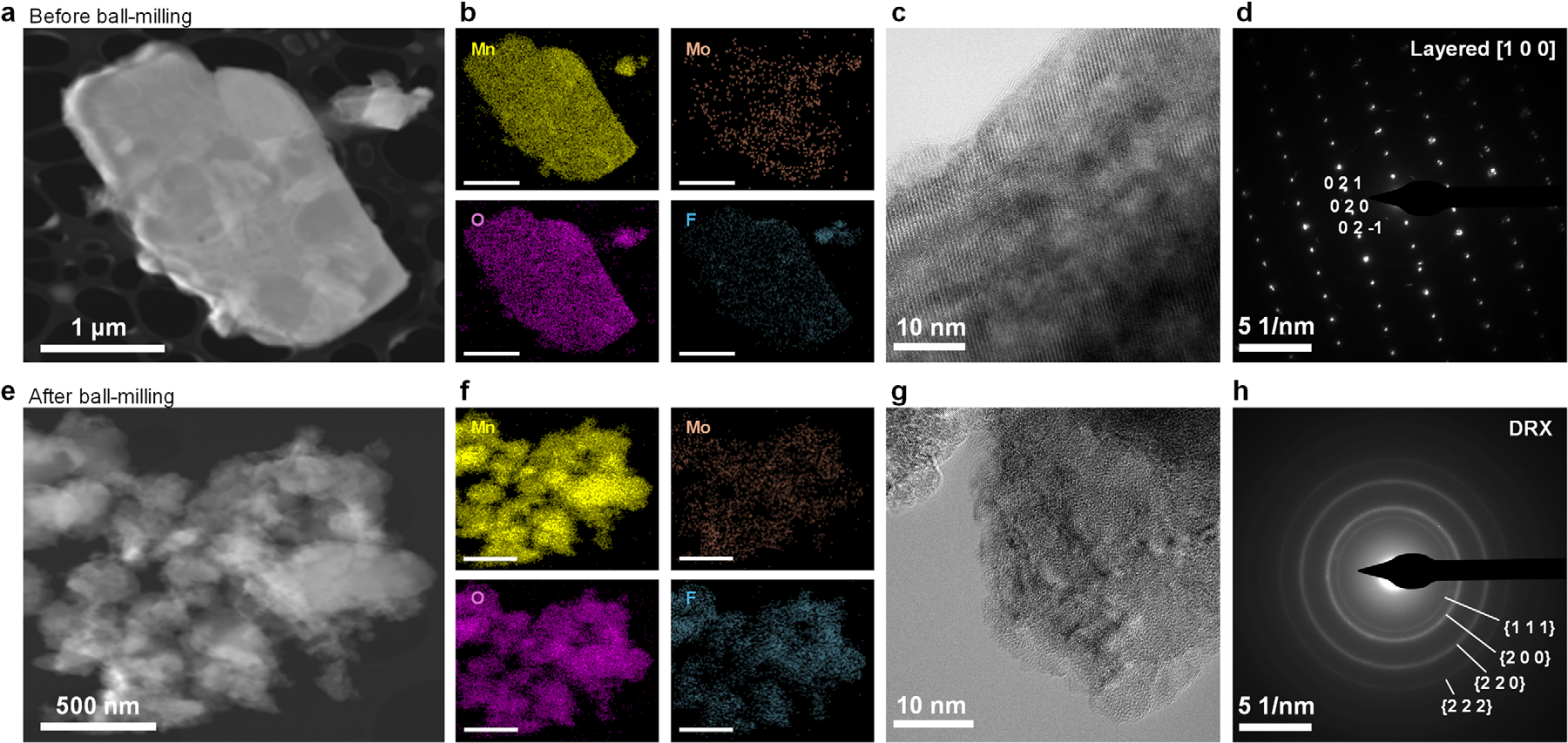
Transmission electron microscopic images of LMMOF0.2. (a,e) HAADF‐STEM images, (b,f) EDS elemental mapping, (c,g) High‐resolution TEM images, and (d,h) SAED patterns of before and after ball‐milling, respectively.

The morphology and atomic‐scale structural evolution induced by mechanochemical transformation were investigated using scanning electron microscopy (SEM) and high‐angle annular dark‐field scanning transmission electron microscopy (HAADF‐STEM). Prior to ball‐milling, the LMMOF0.2 particles appear as irregular micrometer‐sized agglomerates with dimensions of approximately 5–20 µm (Figure 4a; Figures S14, and S15a). After ball‐milling, these particles are transformed into irregularly shaped nanoparticles with sizes ranging from 20 to 50 nm, resulting from particle fracture, deformation, and restructuring under high localized pressure and temperature (Figure 4e; Figures S14, and S15b). The mechanochemical process also significantly increases the Brunauer–Emmett–Teller (BET) surface area of the materials from 0.19 to 11.76 m^2^ g^−1^, offering a larger contact area between active materials and electrolyte (Figure S16). Energy‐dispersive X‐ray spectroscopy (EDS) mapping reveals the elemental distributions before and after ball‐milling (Figure 4b,f). Before ball‐milling, the elemental distribution, particularly of Mo and F, is relatively non‐uniform, whereas a more homogeneous distribution is observed after ball‐milling. Notably, no distinct Mo‐based crystalline phases, such as the phenakite‐type Li_2_MoO_4_ phase observed prior to ball milling, are detected in the XRD patterns after ball milling. Combined with the uniform Mo distribution revealed by EDS mapping, this suggests that Mo is either incorporated into the DRX lattice or exists as a highly dispersed amorphous state, rather than forming segregated secondary phases.

High‐resolution transmission electron microscopy (TEM) images further illustrate the structural transformation, showing large‐grained particles with layered lattice fringes before ball‐milling and significantly reduced grain sizes with DRX lattice fringes after ball‐milling (Figure 4c,g). Selected‐area electron diffraction (SAED) patterns of the pristine sample display predominantly single‐crystalline spot patterns with discernible defects, which may be associated with the phenakite structure (Figure 4d; Figure S17a). In contrast, the ball‐milled particles exhibit ring SAED patterns, consistent with polycrystalline DRX structures (Figure 4h; Figure S17b) [39, 40].

### 2.3 Computational Simulations of Mechanochemical Transformation

**FIGURE 5:**
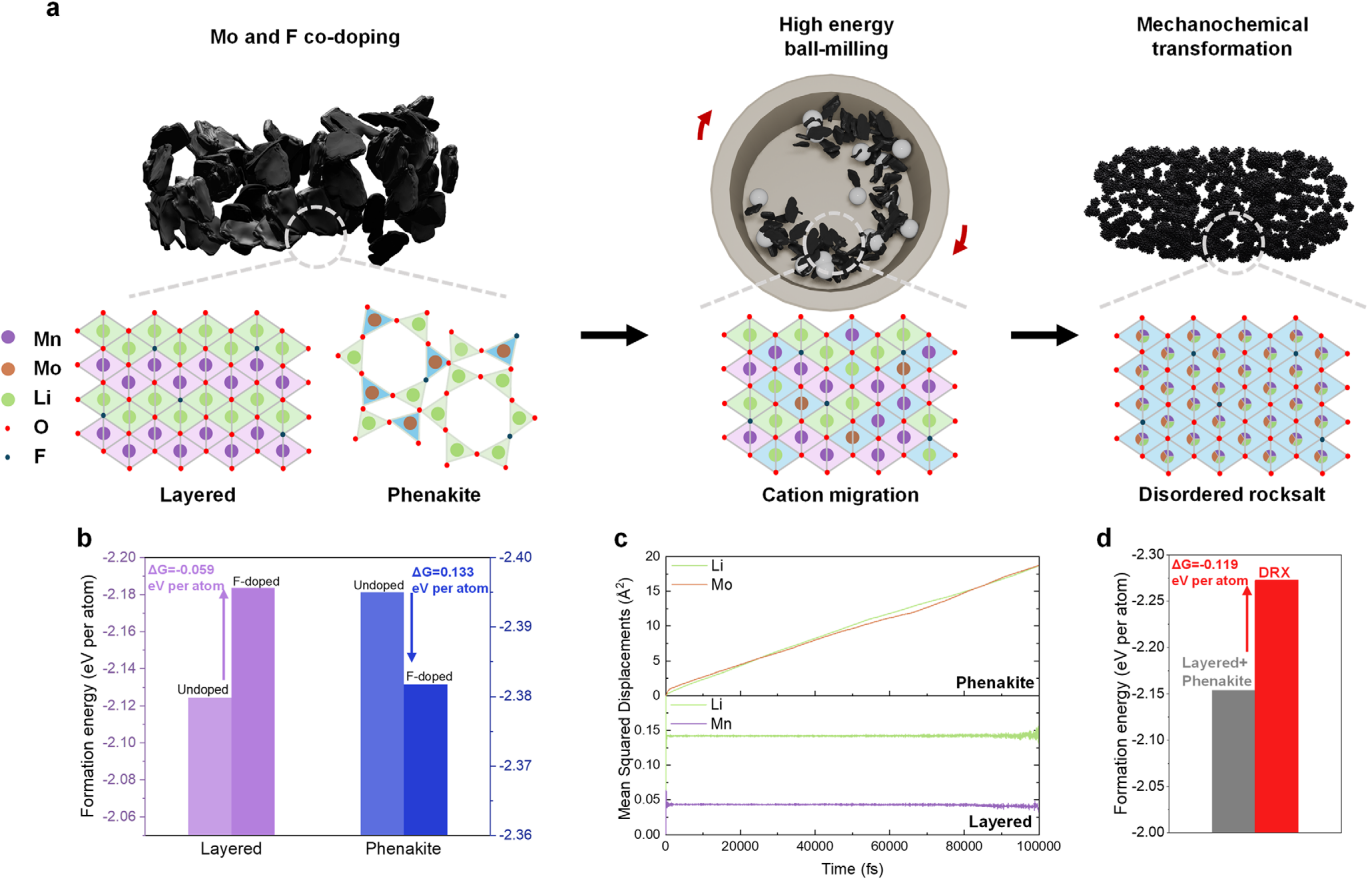
Computational simulation of mechanochemical synthesis from layered/phenakite‐mixed phase to DRX phase. (a) Schematic illustration of structural formation during ball‐milling. (b) Influence of F‐doping on the formation energies of layered and phenakite phases from DFT simulation. (c) Mean square displacement from AIMD simulations of the phenakite and layered structures. (d) Formation energies of the layered/phenakite‐mixed phase and DRX phase from DFT simulation. The layered/phenakite‐mixed phase is modeled as a mechanical mixture of the two phases rather than an explicit heterogeneous interface.

The mechanochemical synthesis process from the layered/phenakite‐mixed phase to the DRX phase was investigated using density‐functional theory (DFT) and *ab initio* molecular dynamics (AIMD) to elucidate the thermodynamic and kinetic origins of ball‐milling‐induced phase transition. As indicated by the XRD and Raman analyses, F doping promotes the formation of a layered/phenakite‐mixed phase in the as‐prepared material. Under ball‐milling conditions, this layered/phenakite‐mixed phase transforms into the DRX structure, whereas a layered‐only structure shows minimal structural evolution under the same conditions, suggesting higher structural stability (Figure 5a). To quantify the effect of F doping on phase stability, formation energies of the layered and phenakite structures were calculated for both undoped and F‐doped configurations (Figure 5b; Figure S18a–d, Note S2). These calculated energies represent relative thermodynamic stability within the DFT framework and do not include temperature‐dependent free‐energy contributions under non‐equilibrium ball‐milling conditions. F doping increases the formation energy of the layered structure while decreasing that of the phenakite structure, indicating that F doping thermodynamically favors the formation of the layered/phenakite‐mixed phase.

The transformation from the layered/phenakite‐mixed phase to the DRX structure requires cation migration initiated at the grain surfaces, which can be activated by the localized pressure and heat generated during ball‐milling. To capture this effect, cation migration in F‐doped layered and phenakite structures was simulated using AIMD at temperatures of 673 and 1073 K (Figure 5c; Figure S19, Note S3). In this work, the layered/phenakite‐mixed phase is modeled as a mechanical mixture rather than a heterogeneous interface. This is because, under ball‐milling conditions, interfacial contacts are continuously generated and disrupted by localized pressure and impact, leading to highly dynamic and non‐equilibrium interfaces that cannot be uniquely defined within a static DFT framework. Constructing a specific interface model would therefore introduce structural bias depending on the chosen configuration. Instead, by separately evaluating the cation migration behavior in each phase, we capture the intrinsic mobility differences that govern interfacial cation exchange during mechanochemical transformation. The results reveal enhanced cation migration of both Li and Mo in the phenakite structure, whereas Li and Mn in the layered structure show negligible migration under identical conditions. This pronounced contrast in intrinsic cation mobility provides the driving force for interfacial cation exchange during transient contact events under ball milling, thereby promoting the formation of the DRX lattice [41].

The calculated formation energy for the transformation from the layered/phenakite‐mixed phase to the DRX phase is approximately −0.119 eV per atom, indicating that the DRX phase is thermodynamically accessible under mechanochemical synthesis conditions (Figure 5d; Figure S18e).

The combined XRD, Raman, XPS, XANES, and EXAFS analyses demonstrate that Mo and F co‐doping plays a key role in promoting the mechanochemical transition from a layered/phenakite‐mixed phase to a DRX phase. XRD and Raman reveal that Mo and F co‐doped LMMOF0.2 primarily converts to DRX under ball‐milling, driven by a synergistic reduction of the cation migration energy barrier. XANES and XPS analyses reveal slight Mn and Mo oxidation and suggest a shift from TM─O─F environments toward increased TM‐F coordination, which more strongly withdraws electron density and correlates with the reduction of non‐lattice oxygen. EXAFS further confirms shortened TM‐O/F and TM‐TM distances, including a significant Mn‐TM contraction associated with cation rearrangement with disorder. Complementary DFT and AIMD simulations provide mechanistic insight into this transition, showing that F doping destabilizes the layered structure while stabilizing the phenakite phase and yields a sufficiently low formation energy for the layered/phenakite‐mixed phase. Enhanced cation mobility within the F‐doped phenakite structure facilitates interfacial cation exchange, thereby promoting DRX formation during ball‐milling. Together, these findings elucidate how the layered/phenakite‐mixed phase evolves into a DRX phase through a mechanochemically driven transformation.

### 2.4 Influence of Mechanochemical Transformation on Electrochemical Properties

**FIGURE 6:**
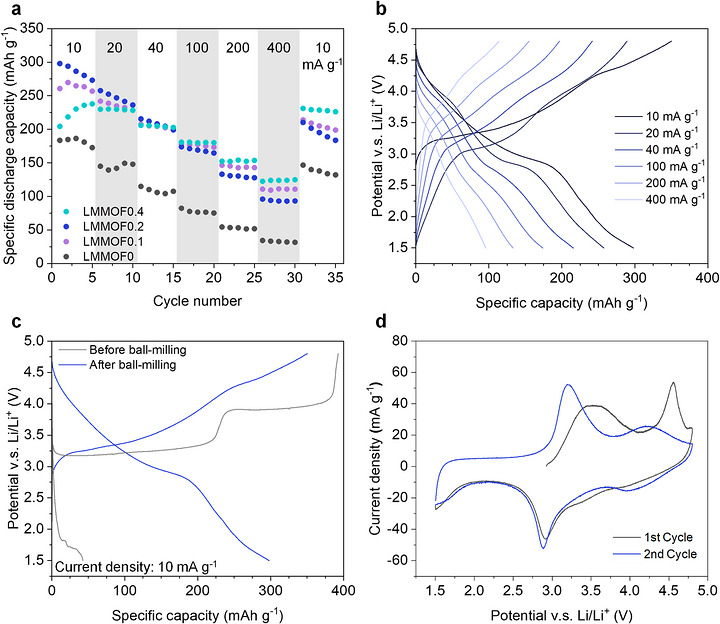
Electrochemical performance of LMMOF electrodes. (a) Rate capabilities of LMMOF0, LMMOF0.1, LMMOF0.2, and LMMOF0.4 at various current densities, ranging from 10 to 400 mA g^−1^. All samples were ball‐milled under the same conditions. (b) Voltage profiles of LMMOF0.2 after ball‐milling at different current densities. (c) First cycle voltage profiles of LMMOF0.2 before and after ball‐milling. (d) First and second CV curves of LMMOF0.2 after ball‐milling at a scan rate of 0.05 mV s^−1^.

To evaluate the electrochemical properties of the ball‐milled LMMOF electrodes, CR2032 coin cells were assembled and tested. The rate capability measurements were performed at current densities ranging from 10 to 400 mA g^−1^ within a voltage window of 1.5 – 4.8 V (Figure 6a, b; Figure S20). Among the samples, LMMOF0.2—dominated by the DRX phase—exhibited the highest reversible specific discharge capacity, delivering 297.9 mAh g^−1^ at a low current density of 10 mA g^−1^. This value is significantly higher than those of LMMOF0, LMMOF0.1, and LMMOF0.4, which contain lower fractions of the DRX phase. However, consistent with the intrinsically sluggish Li^+^ diffusion typically associated with DRX materials, the capacity of LMMOF0.2 decreases rapidly at higher current densities [42]. In contrast, LMMOF0, which is primarily composed of an ordered rocksalt phase, exhibits consistently inferior rate performance across the entire current density range. Additionally, the charge‐transfer resistance (R_ct_) is governed not only by the F content but also by the phase evolution, progressively restricting Li transport from rocksalt‐containing LMMOF0 to predominantly DRX phases (LMMOF0.1 and LMMOF0.2), and ultimately to the layered/DRX composite structure in LMMOF0.4 (Figure S21, Note S4).

The phase transformation also plays a critical role in the electrochemical behavior of LMMOF0.2. Before ball‐milling, LMMOF0.2 shows an initial charge capacity of 392.7 mAh g^−1^ with two distinct two‐phase redox plateaus at approximately 3.2 V and 4.0 V, corresponding to Mn^3+^/Mn^4+^ and O^−^/O^2−^ redox couples, respectively (Figure 6c) [43]. These plateaus are indicative of potential phase separation behavior in the layered/phenakite‐mixed phase during initial delithiation. However, the two‐phase reactions are largely irreversible, which correlates with the observed severe capacity fading and poor reversible capacity of 42.6 mAh g^−1^ upon discharge. After ball‐milling, LMMOF0.2 instead exhibits a single‐phase redox process during both charge and discharge, characterized by smooth voltage slopes and significantly improved reversible capacity. In addition, before ball‐milling, the R_ct_ value increases significantly after the first formation cycle, while the ball‐milled sample exhibits only a slight change (Figure S22). This behavior suggests that the DRX phase generated through mechanochemical activation suppresses irreversible phase transitions and stabilizes the redox reactions, thereby enabling reversible Li^+^ insertion and extraction.

Despite adopting a DRX structure after ball‐milling, LMMOF0.2 undergoes notable electrochemical evolution between the first and second cyclic voltammetry (CV) cycles (Figure 6d). During the first anodic scan, redox activity is observed only above 3.0 V, where high‐voltage redox processes dominate and trigger local structural rearrangements within the DRX framework. Although this activation does not significantly alter the total amount of redox‐active species—as evidenced by the comparable capacities in the first and second cycles—it implies a reorganization of the reaction pathway. The first CV cycle is characterized by a delayed anodic onset and broad, asymmetric features, whereas the second cycle exhibits an earlier current onset, sharper redox peaks, and reduced polarization, reflecting enhanced reaction kinetics and a more stabilized redox mechanism. This activation‐induced opening of the low‐voltage Mn redox pathway accounts that the first charge proceeds only above 3.0 V, while subsequent cycles initiate at much lower potential (∼1.5 V) despite delivering similar overall capacities.

### 2.5 Structural Evolution Under Different Voltage Windows

**FIGURE 7:**
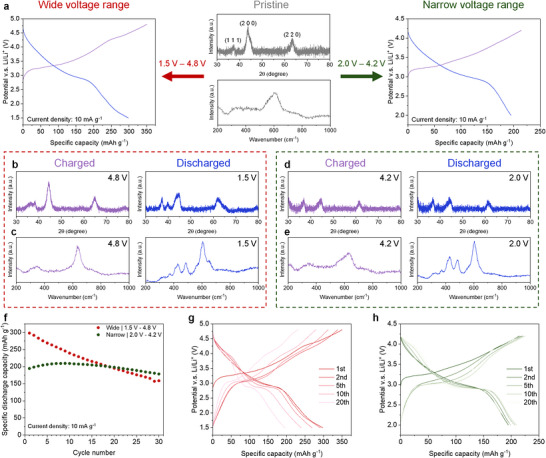
Structural and electrochemical behavior of LMMOF0.2 under wide and narrow voltage ranges. (a) XRD pattern and Raman spectrum of the pristine electrode and its initial cycle voltage profiles with wide and narrow voltage ranges. XRD patterns at charged and discharged states with (b) wide and (c) narrow voltage ranges. Raman spectra at charged and discharged states with (d) wide and (e) narrow voltage windows. (f) Specific discharge capacities for long‐term cycling at a current density of 10 mA g^−1^ under wide and narrow voltage ranges and (g, h) corresponding voltage profiles.

To investigate the effect of voltage window on the structural and electrochemical properties of the LMMOF0.2 electrode, long‐term cycling tests were conducted using a wide voltage range (1.5 – 4.8 V) and a narrow voltage range (2.0 – 4.2 V). Pristine electrodes were initially charged and discharged within each voltage range at a current density of 10 mA g^−1^ (Figure 7a). In the first cycle, the electrodes delivered specific discharge capacities of 297.9 and 194 mAh g^−1^ in the wide and narrow voltage ranges, respectively, corresponding to initial coulombic efficiencies of 85.1% and 90.9%.

For the wide voltage range, the (111), (200), and (220) reflections in the XRD patterns shifted slightly to higher angles upon charging, consistent with lattice contraction of the DRX phase during delithiation (Figure 7b). Although these peaks largely reverted to lower angles after discharge, partial retention at higher angles suggests the presence of irreversible lattice distortion. A weak additional peak is also observed near 40° in the discharged state, indicating that minor local structural rearrangements may occur upon lithiation. Raman spectroscopy further reveals a slight shift of the Mn‐O stretching mode (∼600 cm^−1^) to higher wavenumbers, along with increased intensity in the charged state, reflecting strengthened Mn‐O interactions associated with shortened bond lengths (Figure 7c). Upon discharge, the Mn‐O stretching mode returns to ∼600 cm^−1^, accompanied by the appearance of Mn‐O‐Mn deformation and Mn‐O bending modes at ∼440 and ∼490 cm^−1^, respectively. These Raman features suggest a local reorganization of MnO6 octahedra within the Mn‐rich rocksalt/DRX framework, rather than the formation of a fundamentally different bulk structure. This interpretation is further supported by the weak additional XRD peak near 40° observed in the discharged state, which likely originates from locally rearranged Mn‐rich domains with increased short‐range ordering or distortion. The persistence of higher‐wavenumber features in the Mn‐O stretching vibration supports the occurrence of irreversible local bonding changes.

In contrast, within the narrow voltage range, the (111), (200), and (220) peaks exhibited negligible shifts upon charging, although the (200) peak exhibited a slight decrease in intensity, reflecting limited delithiation (Figure 7d). No significant changes in peak position were observed after discharge, suggesting a near‐zero‐strain structural response. Raman spectra revealed only a slight shift of the Mn‐O stretching mode to higher wavenumbers during charging, with minimal intensity variation (Figure 7e). The peak broadening of the Mn‐O stretching mode is preserved, indicating that the disordered structure remains unchanged during charging. Upon discharge, the Mn‐O stretching mode fully returns to ∼600 cm^−1^ with a reduced bandwidth, suggesting partial relaxation of local structural distortion upon lithiation, while the overall DRX framework is maintained. In addition, the minor emergence of Mn‐O‐Mn deformation and Mn‐O bending modes is observed at ∼440 and ∼490 cm^−1^, respectively. In contrast to the wide‐voltage range, no irreversible Mn‐O stretching was observed.

As a result, the structural degradation observed under the wide voltage range correlates with poorer cycling stability, whereas the narrow voltage range promotes more stable capacity retention (Figure 7f). After 30 cycles at 10 mA g^−1^, the LMMOF0.2 electrode retained 53.3% of its initial capacity in the wide voltage range and 91.7% in the narrow voltage range. This trend is consistent with the voltage profiles (Figure 7g,h), which show stable electrochemical behavior within the narrow voltage range but progressive degradation under the wide voltage range.

## 3 Conclusion

In this study, we demonstrate a mechanochemically driven transformation from a zigzag‐type layered/phenakite‐mixed phase to a DRX structure in Mn‐rich LMMOF materials, enabled by Mo and F co‐doping. Comprehensive structural analyses reveal that while F incorporation strengthens local TM─F bonding, it thermodynamically destabilizes the formation of the layered phase by increasing its formation energy and stabilizes the phenakite phase. This energetic shift promotes the formation of a layered/phenakite‐mixed precursor, which exhibits enhanced cation mobility—particularly within the F‐doped phenakite component—thereby lowering the mechanochemical disordering barrier during ball‐milling. The resulting DRX‐dominant LMMOF0.2 undergoes lattice rearrangement, including shortened TM‐O/F and TM‐TM distances, reflecting increased cation disorder and local structural reconstruction. Electrochemically, mechanochemical activation suppresses the irreversible two‐phase Mn‐ and O‐redox behavior observed in the pristine material and enables a reversible single‐phase redox process with a high capacity of 297.9 mAh g^−1^. Voltage‐dependent structural studies reveal that wide‐voltage cycling induces irreversible lattice distortion in the DRX framework, whereas restricting the voltage range preserves a near‐zero‐strain response and enhances cycling stability. Overall, this study provides a mechanistic understanding of how mixed‐phase precursors modulate phase stability, cation mobility, and disorder formation during mechanochemical synthesis, establishing a generalizable design strategy for developing high‐capacity, earth‐abundant Mn‐rich DRX cathodes with tunable disorder and improved electrochemical performance.

## 4 Experimental Section/Methods

### 4.1 Materials Synthesis

Li_1.2_Mn_(2+x)/3_Mo_(0.4‐x)/3_O_2‐x_F_x_ (*x* = 0, 0.1, 0.2, 0.4) were synthesized using the solid‐state synthesis method. Li_2_CO_3_ (Sigma Aldrich, 99.9%), Mn_2_O_3_ (Sigma Aldrich, 99.9%), MoO_3_ (Thermo Fisher, 99.5%), LiF (CERAC, Inc. 99.9%) were ground together and calcined at 1000°C for 12 h at a ramping rate of 5°C min^−1^ under 10 mL min^−1^ argon flow in a quartz tube furnace and then cooled down to room temperature. Stoichiometric amounts of the precursors were used, with the exception of a 5% mol of excess Li added as Li_2_CO_3_ to compensate for Li loss during the calcination step. After calcination, the resulting powder was sealed in ball‐milling containers in an argon‐filled glovebox and ball‐milled for 4 h at 450 rpm using a U.S. Stoneware Ball Mill.

### 4.2 Electrochemical Measurements

Half‐cells were used for electrochemical evaluation. The working electrode consisted of LMMOFx as the active materials, Super P (TIMCAL) as the conductive additive, and polytetrafluoroethylene (PTFE) as the binder, mixed at a mass ratio of 7:2:1. Electrodes were fabricated via a dry process without adding drying steps. The diameter of the electrode was 10 mm, with an active material mass loading of 3.0 – 4.0 mg cm^−2^. A 1.0 M LiPF_6_ in ethylene carbonate/dimethyl carbonate (EC/DMC, 1:1 v/v) solution (Sigma–Aldrich) was used as the electrolyte, and glass microfiber (Whatman) served as the separator. Li metal (MTI) was used as both the reference and counter electrode. Electrochemical measurements were conducted using CR2032 coin cells assembled in an argon‐filled glovebox to ensure a controlled testing environment.

Galvanostatic charge–discharge measurements were conducted using Neware battery testers (CT4008) at various current densities to evaluate the electrochemical performance of the as‐prepared cells. The cyclic voltammetry was performed at a defined scan rate using an Ametek potentiostat. The electrochemical impedance spectroscopy (EIS) measurements were performed using the same potentiostat over a frequency range of 0.01 to 10 000 Hz with an *ac* voltage amplitude of 10 mV at open‐circuit voltage. All electrochemical measurements were performed at room temperature.

### 4.3 Thermogravimetric Analysis and Differential Scanning Calorimetry

Samples were heated from 25 to 1100°C at a ramp rate of 5°C min^−1^ under an argon atmosphere with a flow rate of 10 mL min^−1^ using a TA Q600 SDT instrument.

### 4.4 X‐ray Diffraction

XRD patterns were obtained using Rigaku Miniflex Powder XRD with a Cu K𝛼 radiation source. The 2𝜃 range was 10°–80°.

### 4.5 X‐ray Photoelectron Spectroscopy

XPS spectra were obtained using Thermo K‐alpha XPS.

### 4.6 Raman Measurement

Raman spectroscopy was performed using a Renishaw RM1000 microspectroscopic system with a 50x objective and an Ar laser excitation (514 nm). Every measurement was repeated at least three times to obtain average profiles.

### 4.7 Scanning Electron Microscopy

SEM images were obtained using a Hitachi SU8230SEM with a beam voltage of 5–10 kV.

### 4.8 Transmission Electron Microscopy and Energy Dispersive X‐ray Spectroscopy

The TEM images were obtained using FEI Tecnai G2 F30. EDS was performed at a beam voltage of 200 kV.

### 4.9 Specific Surface Area Analysis

The surface area was measured by Micromeritics TriStar II surface and Porosity Analyzer through the BET method.

### 4.10 Computational Methods

All spin‐polarized calculations were performed with the density functional theory method using the Vienna ab initio simulation package (VASP) [44, 45]. The projector augment wave (PAW) method was applied with Mo([Ar]4s^2^4p^6^4d^5^5s^1^), Mn([Ne]3p^6^3d^5^4s^2^), F([He]2s^2^2p^5^), O([He]2s^2^2p^4^), Li(1s^2^2s^1^), to solve the interaction between ionic core electrons and valence electrons. The generalized gradient approximation (GGA) with Perdew—Butke–Ernzerhof (PBE) functional was used to take the exchange‐correlation into consideration in the Kohn–Sham equations [46]. The energy cutoff and convergence criteria were set as 520 and 10^−5^ eV, respectively. Gamma k‐point meshes were generated to achieve a reciprocal‐space resolution of 0.04–0.05 Å^−1^, consistent with high‐throughput DFT protocols used in the Materials Project [47]. The structures were relaxed until the force on each atom was less than 0.02 eV Å^−1^. The RMM‐DIIS algorithm and the conjugate‐gradient were used during the electronic and ionic optimization, respectively. The dispersion correction, DFT‐D3, is also considered.

Ab initio molecular dynamics simulations were performed in VASP using an on‐the‐fly machine‐learning force field (MLFF). The PAW method and PBE exchange–correlation functional were used throughout. All pristine and F‐doped layered and phenakite structures were fully relaxed prior to MD simulations.

## Author Contributions

Y. A. conceived the research idea and designed the experiments. Y.‐C. W. synthesized the materials. Y.‐C. W. and Y. A. carried out the electrochemical measurement and physicochemical characterizations. T.‐Y. C. performed XAS measurements. X. H. conducted the DFT and AIMD simulations. Y. D. carried out the TEM analysis. Y. O. contributed to scientific discussions and prepared illustrations. W. W. performed the BET measurements. Y.‐C. W., Y. A., and M. L. engaged in data analysis and scientific interpretation. Y.‐C. W. and Y. A. drafted the manuscript, and M. L. revised and finalized it. All authors reviewed and approved the final version of the manuscript.

## Funding

This research was supported by the Hightower Endowment through the Georgia Tech Foundation.

## Conflicts of Interest

The authors declare no conflicts of interest.

## Supporting information




**Supporting File**: smll73666‐sup‐0001‐SuppMat.docx.

## Data Availability

All data supporting the findings of this study is available from the authors upon request.
