# Supplementary material for: Mechanochemical Transformation From Zigzag‐Type Layered/Phenakite to Disordered Rocksalt in Mn‐Rich Cathodes for Li‐Ion Batteries
Source: Small. 2026 May 7;22(35):e73666. doi: 10.1002/smll.73666 (PMC13288854; doi:10.1002/smll.73666)
Supplement: Supplementary file 1 — Supporting File: smll73666‐sup‐0001‐SuppMat.docx. [file SMLL-22-e73666-s001.docx]

Supplementary Information

**Mechanochemical Transformation from Zigzag-Type Layered/Phenakite to Disordered Rocksalt in Mn-Rich Cathodes for Li-Ion Batteries**

*Yi-Chen Wu‡, Yoojin Ahn‡, Tsung-Yi Chen, Xueyu Hu, Yong Ding, Yewon Oh, Weining Wang, and Meilin Liu**

‡These authors contributed equally

Y. Wu, Y. Ahn, X. Hu, Y. Ding, Y. Oh, W. Wang, M. Liu

School of Materials Science and Engineering, Georgia Institute of Technology, Atlanta, Georgia 30332-0245, USA

T. Chen

National Synchrotron Radiation Research Center, 101 Hsin-Ann Road, Hsinchu 300092, Taiwan

E-mail: meilin.liu@mse.gatech.edu

Funding: This research was supported by the Hightower Endowment through the Georgia Tech Foundation.

Keywords: lithium-ion battery, cathode, disordered rocksalt, phase transformation, mechanochemical synthesis

Figure S1. Reference X-ray diffraction (XRD) patterns of Li_2_MoO_4_ (phenakite), Li_3_MnO_4_ (Caswellsilverite), LiMnO_2_ (zigzag-type layered), and MnO (rocksalt) structures.

Figure S2. Phase transition analysis of the LMMOF0.2 precursor. (a) Thermogravimetric analysis (TGA) and differential scanning calorimetry (DSC) measurements. TGA and DSC were performed with a ramp rate of 5 °C min^-1^ under an Ar gas flow of 10 mL min^-1^. (b) XRD patterns of LMMOF0.2 calcined at different temperatures ranging from 600 °C to 1100 °C. The calcination was conducted with a ramp rate of 5 °C min^-1^ under an Ar gas flow of 10 mL min^-1^.

Figure S3. Raman bands in the LMMOF samples and schematic illustrations of band examples.

Figure S4. X-ray photoelectron spectroscopy (XPS) plots of Mn 2p in LMMOF samples (a) before and (b) after ball-milling. The Mn^3+^, Mn^4+^, and raw data signals are demonstrated as blue solid lines, purple solid lines, and gray dotted lines, respectively.

Figure S5. X-ray absorption near-edge spectra (XANES) of Mn K-edge of LMMOF samples (a) before and (b) after ball-milling.

Figure S6. XPS plots of Mo 3d in LMMOF samples (a) before and (b) after ball-milling. The Mo^5+^, Mo^6+^, and raw data signals are demonstrated as blue solid lines, purple solid lines, and gray dotted lines, respectively.

Figure S7. XANES of Mo K-edge of LMMOF samples (a) before and (b) after ball-milling.

Figure S8. XPS plots of (a) F 1s and (b) O 1s in the LMMOF0.2 sample. In the F 1s plot, the TM-F, TM-O-F, and raw data signals are demonstrated as blue solid lines, purple solid lines, and gray dotted lines, respectively. In the O 1s plot, the lattice oxygen, non-lattice oxygen, and raw data signals are demonstrated as blue solid lines, purple solid lines, and gray dotted lines, respectively.

Figure S9. Extended X-ray absorption fine structure (EXAFS) of (a, b) Mn K-edge and (c, d) Mo K-edge of LMMOF samples before and after ball-milling.

Figure S10. Extended X-ray absorption fine structure (EXAFS) of Mn K-edge with fitting curves and R-factor values for LMMOF0.2 (a) before and (b) after ball-milling. In the fitting curves, the Mn-TM distance decreases by 11.0% after ball-milling.

Figure S11. XRD patterns of the LMMOF0.2 samples after ball-milling for various times, ranging from 0 to 8 hours.

Figure S12. XPS plots of (a) Mn 2p and (b) Mo 3d in the LMMOF0.2 sample after ball-milling for various times, ranging from 0 to 4 hours. In the Mn 2p plot, the Mn^3+^, Mn^4+^, and raw data signals are demonstrated as blue solid lines, purple solid lines, and gray dotted lines, respectively. In the Mo 3d plot, the Mo^5+^, Mo^6+^, and raw data signals are demonstrated as blue solid lines, purple solid lines, and gray dotted lines, respectively.

Figure S13. (a) XRD patterns and (b) Raman spectra of LMMOF0.2 ball-milled for 2 hours at different rotational speeds of 300, 450, and 600 rpm.


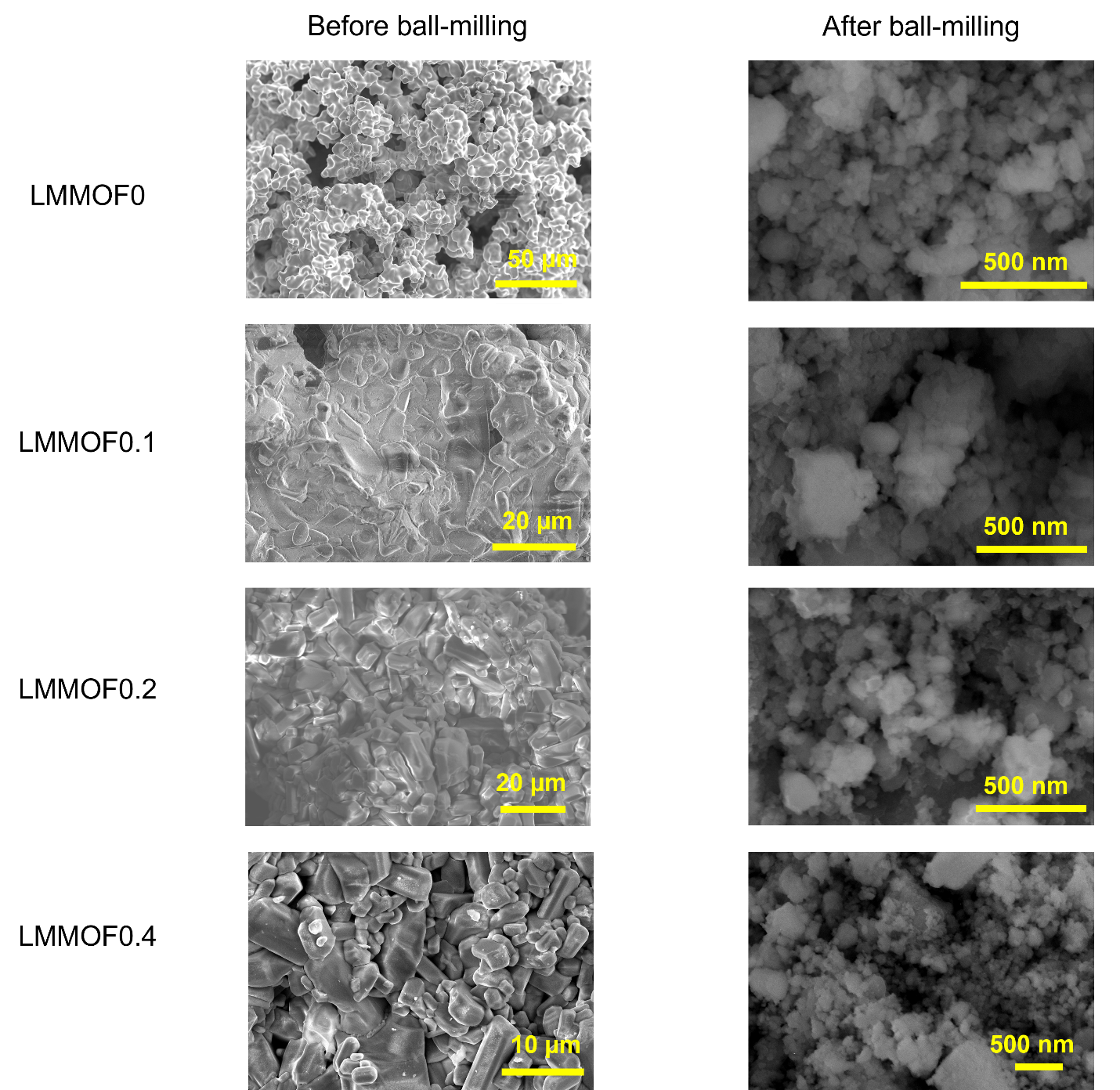


Figure S14. Scanning electron microscopy (SEM) images of LMMOF samples before and after ball-milling.


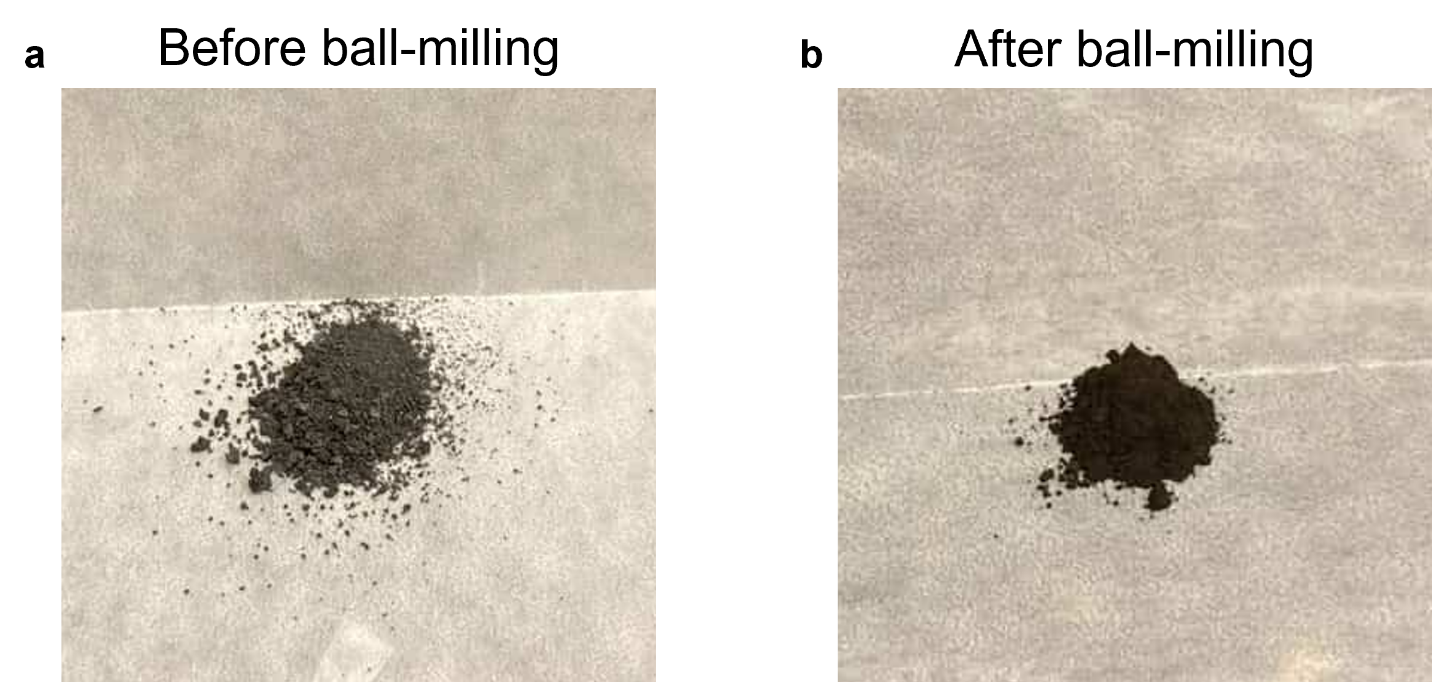


Figure S15. Optical images of LMMOF0.2 powder (a) before and (b) after ball-milling.

Figure S16. BET specific surface area measurements of LMMOF0.2 before and after ball-milling.


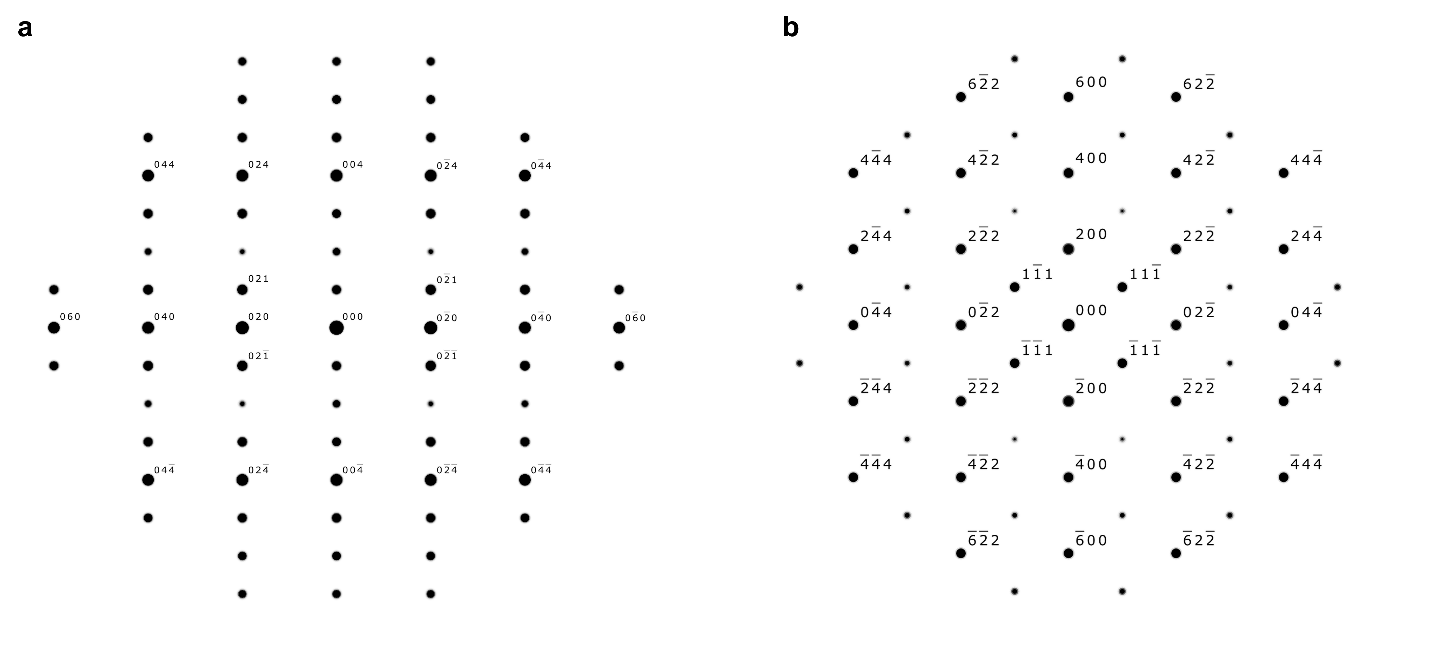


Figure S17. Simulated SAED patterns of (a) layered [1 0 0] and (b) DRX [0 1 1].


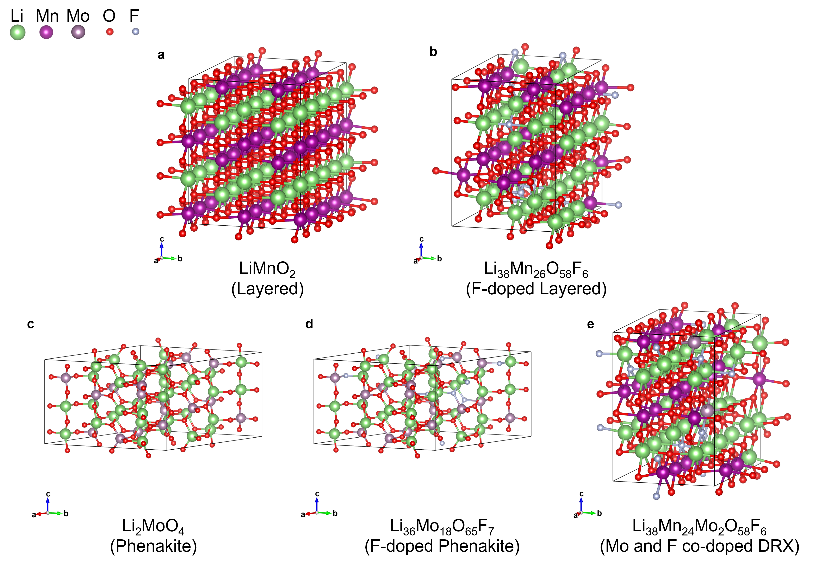


Figure S18. Computational models for density-functional theory (DFT) and molecular dynamics (MD) simulations. (a) Layered LiMnO_2_, (b) F-doped layered Li_38_Mn_26_O_58_F_6_, (c) phenakite Li_2_MoO_4_, (d) F-doped phenakite Li_36_Mo_18_O_65_F_7_, and (e) DRX Li_38_Mn_24_Mo_2_O_58_F_6_. The elemental compositions are calculated based on experimental synthesis.

Figure S19. Mean square displacement from AIMD simulations of (a, b) Layered and (c, d) Phenakite structures at temperatures of 673 K and 1073 K, respectively.

Figure S20. Voltage profiles of (a) LMMOF0, (b) LMMOF0.1, and (c) LMMOF0.4 at various current densities, ranging from 10 to 400 mA g^-1^ under the voltage range of 1.5 – 4.8 V.

Figure S21. Electrochemical impedance spectroscopy (EIS) plots of LMMOF0, LMMOF0.1, LMMOF0.2, and LMMOF0.4 electrodes in the fresh coin cell before cycling.

Figure S22. EIS plots of (a) before and (b) after ball-milling LMMOF0.2 before cycling and after the first cycle at a current density of 10 mA g^-1^.

Supplementary note 1

After ball-milling, the XRD pattern of LMMOF0.2 shows the (1 1 1), (2 0 0), and (2 2 0) reflections at approximately 37.4 °, 43.6 °, and 63.6 °, respectively (Figure 1a). From these peak positions, the lattice parameter of the DRX structure is calculated to be ~4.14 Å. As shown in Figure S1, rocksalt-type MnO exhibits the same reflections at lower angles, corresponding to a larger lattice parameter of ~4.45 Å. The reduced lattice parameter in LMMOF0.2 arises from transition metal-Li cation mixing and the lattice contraction induced by F-doping. Based on the lattice parameter of Li-less MnO rocksalt, we used this reference to identify and define the DRX phases in our XRD analyses.

Supplementary note 2

To investigate the influence of F doping on layered and phenakite precursor phases, supercell models representing the pristine crystal structures were constructed. A 4*a*×2*b*×2*c* supercell of layered LiMnO_2_ containing 64 Li/Mn cations and 64 oxygen atoms was used to model the layered phase. For the phenakite phase, a bulk Li_2_MoO_4_ structure comprising 54 Li/Mo cations and 72 oxygen atoms was employed. Fluorine doping was introduced by substituting 10% of the oxygen sites with fluorine atoms in both structures, enabling a systematic evaluation of the effect of F incorporation on phase stability and formation energetics. To compare the thermodynamic preference between the layered/phenakite precursor mixture and DRX phase, a hypothetical reaction was constructed using bulk models with matched stoichiometry for the reactants and products. Formation energies of all participating phases were calculated independently, and the overall reaction energy difference was obtained to quantify the thermodynamic driving force toward DRX formation. The representative reaction considered is:

$$\frac{9}{5}\mathrm{Li}_{16/27}\mathrm{Mn}_{11/27}O_{122/135}F_{13/135}+\frac{1}{15}\mathrm{Li}_{2}\mathrm{Mo}O_{3.6}F_{0.4}=\mathrm{Li}_{6/5}\mathrm{Mn}_{11/15}\mathrm{Mo}_{1/15}O_{9/5}F_{1/5}+\frac{1}{30}O_{2}(g)$$

All formation energies were rigorously corrected to obtain Gibbs formation energies referenced to 298.15 K. Raw total energies obtained from VASP were converted into corrected formation energies using the *pymatgen* correction framework, following the Materials Project (MP) methodology. This correction scheme systematically compensates for known GGA errors, particularly in transition-metal oxides. For each relaxed structure, the DFT total energy $E_{\mathrm{DFT}}$ was corrected by applying MP anion corrections for oxygen overbinding as well as element-specific cation corrections:

$$E_{\mathrm{corr}}=E_{\mathrm{DFT}}+\sum_{i} n_{i}C_{i}$$

where $n_{i}$ is the number of atoms of element and $C_{i}$ is the corresponding MP correction obtained from *pymatgen.analysis.corrections*. Formation energies were then referenced to MP-standard elemental chemical potentials:

$$\Delta H_{\mathrm{form}}^{\mathrm{corr}}=E_{\mathrm{corr}}-\sum_{i} n_{i}\mu_{i}^{\mathrm{ref}}$$

where $\mu_{i}^{\mathrm{ref}}$ are the fitted elemental reference energies embedded within *pymatgen*. This procedure ensures that all compositions are evaluated on a thermodynamically consistent energy scale compatible with Materials Project phase-diagram construction.

Finite-temperature contributions were estimated using *pymatgen*’s Gibbs energy model, which provides empirical but physically grounded approximations for vibrational and configurational entropy in solid phases. The Gibbs free energy at temperature T was calculated as:

$$G\left( T \right)=\Delta H_{\mathrm{form}}^{\mathrm{corr}}+\Delta G^{\mathrm{solid}}\left( T \right)+n_{O}\frac{1}{2}\Delta\mu_{O_{2}}(T)$$

where $\Delta G^{\mathrm{solid}}\left( T \right)$ accounts for temperature-dependent vibrational and configurational contributions following the quasiharmonic-derived empirical model implemented in *pymatgen*, and $\Delta\mu_{O_{2}}(T)$ is taken from the NIST-JANAF thermochemical tables. This approach avoids computationally expensive phonon calculations while maintaining quantitative reliability consistent with large-scale computational phase-diagram databases.

Supplementary note 3

Ab initio molecular dynamics (AIMD) simulations were performed in VASP using an on-the-fly machine-learning force field (MLFF). The PAW method and PBE exchange–correlation functional were used throughout. All pristine and F-doped layered and phenakite structures were fully relaxed prior to MD simulations.

On-the-fly training (10 ps): Canonical ensemble (NVT) simulations were conducted at 673 K and 1073 K using a Nosé–Hoover thermostat with a time step of 0.5 fs. During the trajectory, configurations exceeding the MLFF uncertainty threshold were recomputed at the DFT level and iteratively added to the training set, enabling continuous refinement of the force field.

Pre-equilibration: After training, the finalized MLFF was frozen. Systems were sequentially equilibrated to remove transient effects. For simulations targeting 673 K, equilibration was performed at 300 K and 673 K for 2.5 ps each. For simulations targeting 1073 K, equilibration was conducted at 300 K, 600 K, and 1073 K for 2.5 ps each, all within the NVT ensemble using a 0.5 fs time step.

Production dynamics: Production runs were carried out in the NVT ensemble at 673 K and 1073 K for 200,000 steps with a 0.5 fs time step (total simulation time of 100 ps). Atomic trajectories and thermodynamic data were recorded for subsequent analysis.

Supplementary note 4

The increase in the charge-transfer resistance (R_ct_) across the LMMOF series cannot be explained solely by F content. Rather, the evolution of the phase formation plays a decisive role, from rocksalt-assisted conduction in LMMOF0, to dominant DRX in LMMOF0.1 and LMMOF0.2, and finally to a heterogeneous layered/DRX mixture in LMMOF0.4. DRX phases exhibit intrinsically limited Li percolation due to random TM distribution, and higher F incorporation further suppresses electronic conductivity through strengthened TM-F bonding. LMMOF, containing both rocksalt and DRX phases, retains relatively accessible Li-ion migration pathways, resulting in the lowest R_ct_. In contrast, the predominant DRX structures in LMMOF0.1 and LMMOF0.2 impose increasingly restricted percolation pathways as cation disorder intensifies, leading to higher R_ct_ compared to LMMOF0. LMMOF0.4 exhibits significantly higher R_ct_ because the presence of a zigzag-type layered structure introduces additional structural anisotropy and phase-boundary resistance, further hindering Li transport relative to the DRX-dominant compositions.
